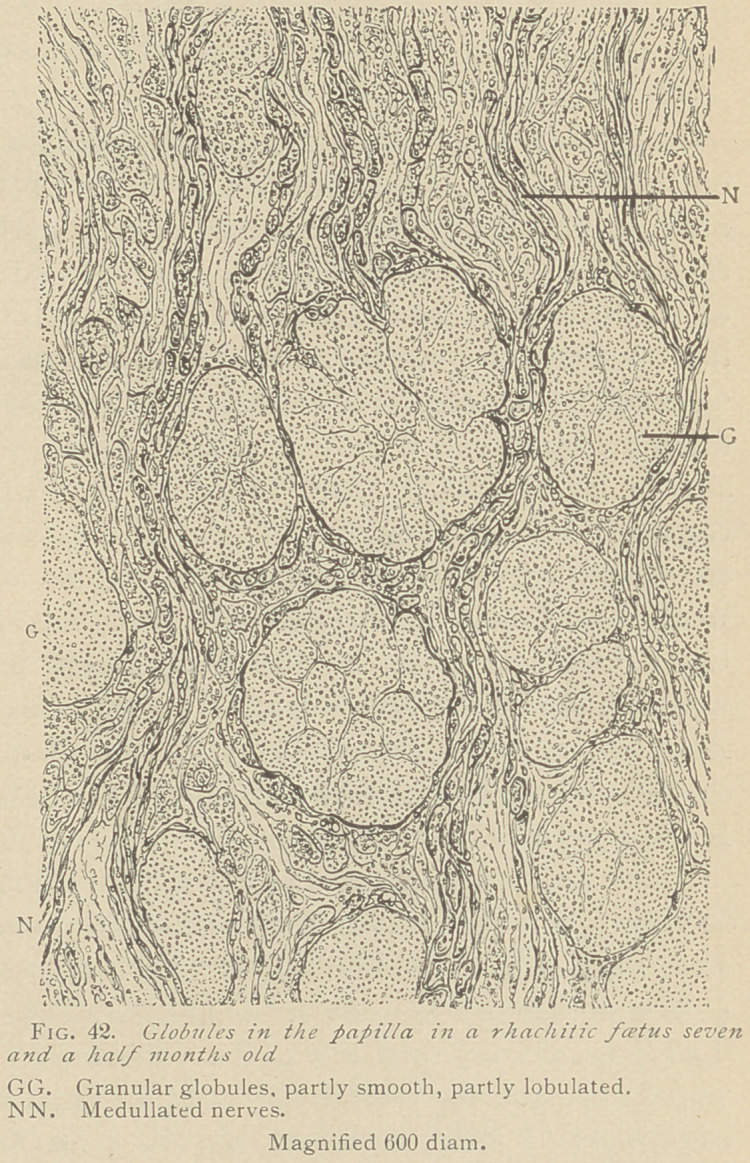# Contributions to the History of Development of the Teeth

**Published:** 1888-01

**Authors:** Carl Heitzmann, C. F. W. Bödecker


### Independent Practitioner.

VOL. IX. JANUARY, 1888. NO. 1.

NOTE.—No paper published or to be published in another journal will be accepted for this department. All papers must be in the hands of the Editor before the first day of the month preceding that in which they are expected to appear. Extra copies will be furnished to each contributor of an accepted original article, and reprints, in pamphlet form, may be had at the cost of the paper, press-work and binding, if ordered when the manuscript is forwarded. The Editor and Publishers are not responsible for the opinions expressed by contributors. The journal is issued promptly, on the first day of each month.

### Original Communications.

### CONTRIBUTIONS TO THE HISTORY OF DEVELOPMENT OF THE TEETH.

BY CARL HEITZMANN, M. D., AND C. F. W. BÖDECKER, D. D. S., M. D. S.

CONTINUED FROM PAGE 625, VOL. VIII.

V. Anomalies of enamel. It is a common feature in teeth of rhachitic children that the interstices between the enamel prisms are wide, and their tenants, the enamel fibers, are very distinct, often running a wavy course independent of the contours of the enamel prisms. The prisms themselves are finely granular, without distinct cross-lines. These features are explained by a deficient calcification of the enamel, which, at the same time, allows the cutting of thin sections of the enamel after it has been softened in chromic acid solution, while it is impossible to obtain sections of normal enamel in the same manner. In a rhachitic fœtus eight months old, the writers have observed dark brown portions in the enamel, which are to be considered as pigmentations of the enamel rods. Another feature in the enamel of rhachitic embryos is a markedly wavy course taken by the enamel prisms, so much so that in a strictly longitudinal section alternate layers of enamel prisms will appear, some of which are cut longitudinally, while others are transverse. (See Fig. 40.)

FIG. 40. *Anomalous enamel of a rhachitic fœtus seven and a half months old.*

EE. Enamel organ of medullary character arisen from the external epithelium.

E. Enamel composed of prisms of a markedly wavy course.

M. Cluster of medullary corpuscles, *i. e.*, non-calcified enamel tissue.

IL. Interzonal layer filled with medullary corpuscles.

D. Dentine.

Magnified 500 diam.

Dr. Frank Abbott has already drawn attention to the fact that transverse sections of enamel prisms, alternating with longitudinal sections, are not caused by an interlacing of the enamel prisms, but by a wavy or devious course of the enamel prisms themselves, and our specimens have furnished satisfactory proof of the correctness of the latter view.

Not infrequently we observe at the summit of the dentine, in the interzonal layer, medullary corpuscles, either arranged in rows or irregularly scattered in the vicinity of the dentine. Such formations scarcely admit of any other interpretation than that the medullary elements from which the enamel prisms originate have not been calcified, but remained in an embryonal state. (See Fig. 40, M. and IL.)

FIG. 41. *Medullary space and globular mass in the dentine of a tooth of a rhachitic fœtus eight months old.*

M. Medullary corpuscles filling a crescentic space in the dentine.

B. Branch of the crescentic space replacing a dentinal canaliculus.

G. Globular concentrically striated mass.

Magnified 600 diam.

In specimens from a rhachitic fœtus seven and a half months old, the enamel appeared bordered by a medullary tissue, whose origin could directly be traced from the buds and clusters, the remains of the external epithelium. Here, therefore, our previous assumption, that the external epithelium likewise furnishes material for the increase of the enamel, can be directly proven.

VI. Anomalies of the dentine. The dentine of all rhachitic teeth is conspicuous by wide dentinal canaliculi, in which the dentinal fibers and their lateral offshoots are easily discernible. The basis-substance shows a more or less marked reticular structure, without the application of any reagent. In a fœtus eight months old the writers found peculiar formations of dentine, which evidently are caused by a deficient calcification of this tissue. (See Fig. 41.)

Such spaces send offshoots upward and downward into the dentine, which represent either conically widened dentinal canals, or broad routes replacing the same. The spaces and their larger branches are filled with medullary corpuscles in all stages of development. Where the space inosculates with dentinal canaliculi, the tenants of the latter are coarse fibrillæ, with spindle-shaped widenings composed of large granules. Both within these spaces and in their vicinity we observe globular formations which exhibit the features of badly developed secondary dentine, or globular dentine, resembling the structure of the dentine of the so-called pulp-stones, or denticles. Spaces of this description resemble the interglobular spaces of Czermak, but they are more irregular and much larger.

VII. Anomalies of the papilla. The writers have described and illustrated, in Fig. 37, anomalous papillæ of peculiar shapes. In such papillæ we often observe crystals of hæmatoidine grouped together in clusters, the origin of which must be sought for in an imbibition by the tissue of the coloring matter of the blood at a very early stage of development. The writers furthermore wish to draw attention to peculiar formations met with in the medullary tissue of the papilla. (See Fig. 42.)

Such globules we have observed only at the summit of the papilla, and in close connection with medullated nerve fibers. The globules are pale, finely granular, and with either smooth or lobulated contours. Their interior shows faint marks of division, which indicate that the globules have arisen from medullary corpuscles, or clusters thereof. We are unable to determine the nature of such corpuscles, which seem to be in relation to newly forming medullated nerves. All the nerve fibers seem, however, to run between the globules, although it appears in the drawing as if a bundle of nerves inosculated with a globule. This may be explained by a devious course of the nerve bundle.

FIG. 42. *Globules in the papilla in a rhachitic fœtus seven and a half months old*

GG. Granular globules, partly smooth, partly lobulated.

NN. Medullated nerves.

Magnified 600 diam.

The most interesting feature of such papillæ is that the medullated nerves first appear at the summit of the papilla, whereas the lower portions of the papillæ are free from nerves, and only exhibit scanty capillary blood-vessels. The nerves still appear to be composed of rows of medullated corpuscles, without any trace of the myeline sheath. Whether or not axis cylinders are present the writers could not determine in the longitudinal sections before them. This much is certain—that the nerves grew independent of the central nervous system; that they were in no connection with already formed nerves of the central nervous system, and, therefore, that they must have arisen from medullary corpuscles in essentially the same manner as other tissues.

Those who strictly adhere to the doctrine of exclusiveness in embryology, depending upon the three original layers, will try in vain to explain such an independent formation of nerves in the middle of connective tissue.

(TO BE CONTINUED.)

## Figures and Tables

**Fig. 40. f1:**
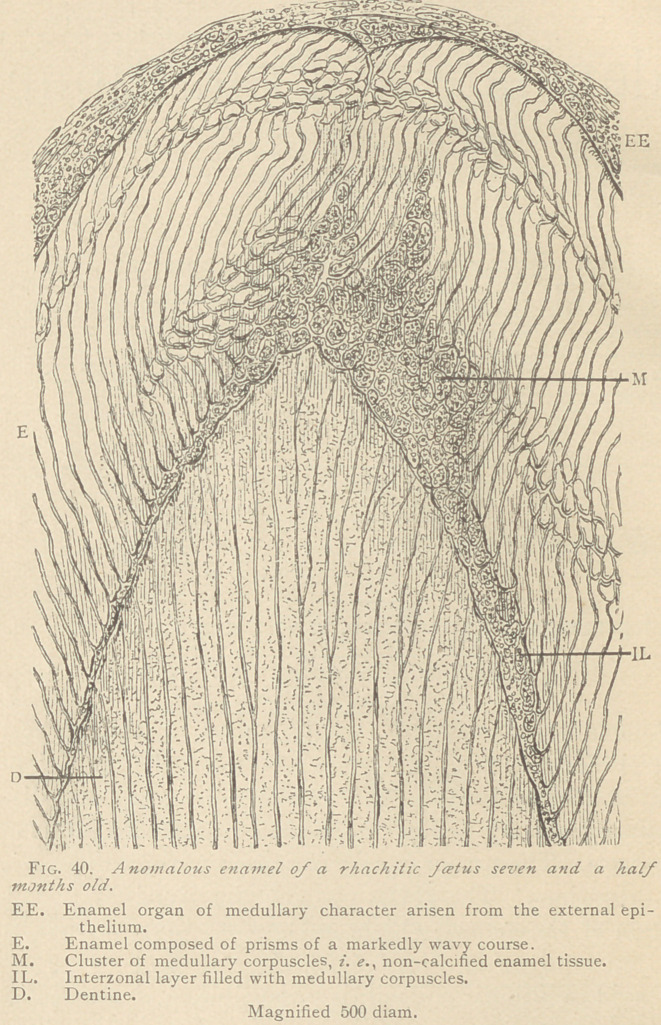


**Fig. 41. f2:**
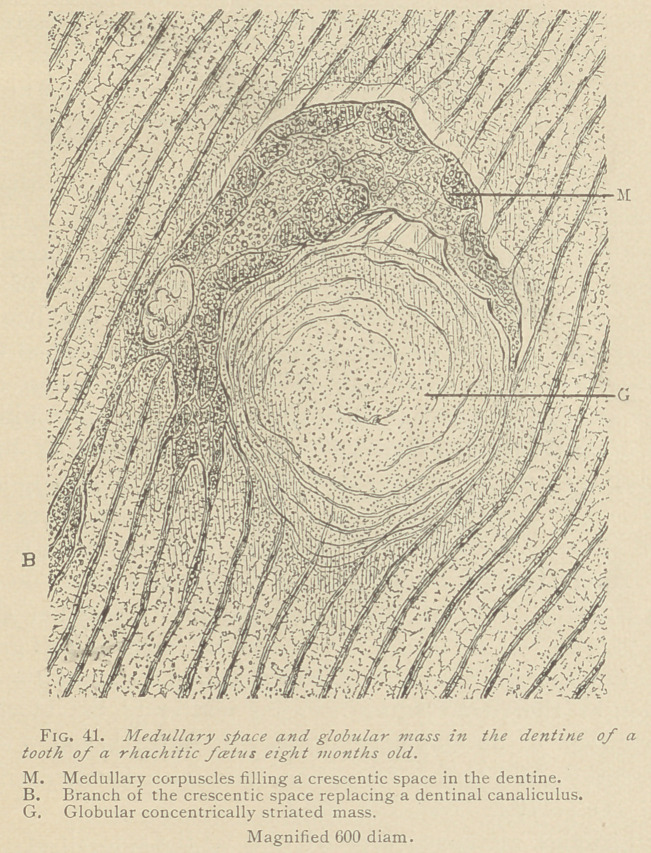


**Fig. 42. f3:**